# Supplementary material for: Thermoregulatory, Cardiovascular and Perceptual Responses of Spectators of a Simulated Football Match in Hot and Humid Environmental Conditions
Source: Sports (Basel). 2023 Mar 31;11(4):78. doi: 10.3390/sports11040078 (PMC10140829; doi:10.3390/sports11040078)
Supplement: Supplementary file 1 [file sports-11-00078-s001.zip › sports-2291141-supplementary.pdf]

Supplementary Table S1. Sex differences in thermal and cardiovascular outcome parameters

	Total group (n=48)	Males (n=30)	Females (n=18)	P-value
Age (years)	43 ± 19	47 ± 18	36 ± 18	0.04
Baseline Tcore (°C)	37.3 ± 0.4	37.1 ± 0.7	37.5 ± 0.3	<0.004
Peak Tcore (°C)	37.5 ± 0.3	37.4 ± 0.3	37.6 ± 0.3	0.10
ΔTcore (°C)	0.2 ± 0.2	0.3 ± 0.0	0.1 ± 0.1	0.001
Baseline Tskin (°C)	32.8 ± 0.8	32.9 ± 0.9	32.9 ± 0.6	0.92
Peak Tskin (°C)	35.4 ± 0.3	35.4 ± 0.3	35.5 ± 0.3	0.67
ΔTskin (°C)	2.6 ± 0.8	2.5 ± 0.9	2.6 ± 0.6	0.80
Baseline HR (bpm)	76 ± 15	72 ± 16	81 ± 11	0.041
Peak HR (bpm)	81 ± 14	78 ± 14	87 ± 12	0.05
ΔHR (bpm)	6 ± 7	6 ± 8	4 ± 5	0.28
Baseline MAP (mmHg)	96 ± 8	100 ± 10	93 ± 9	0.017
Peak MAP (mmHg)	100 ± 8	103 ± 9	97 ± 10	0.033
ΔMAP (mmHg)	4 ± 5	3 ± 4	4 ± 5	0.58

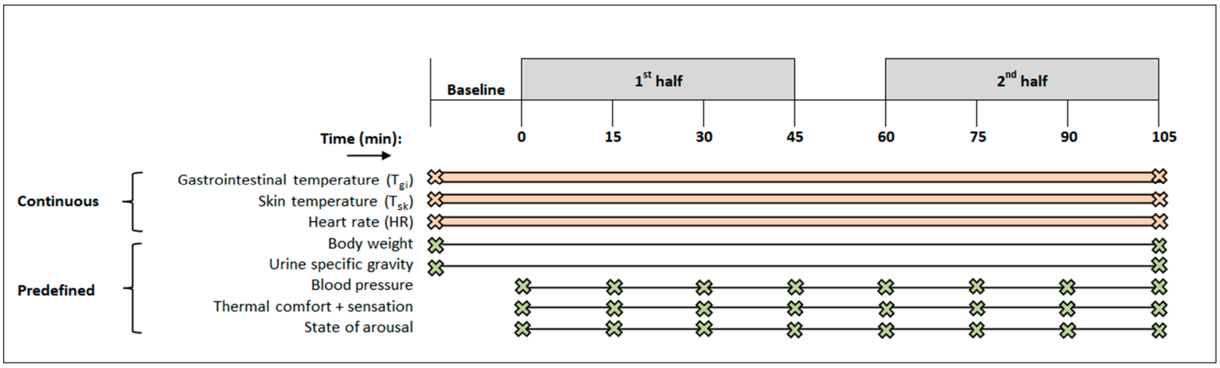

Supplementary Figure S1. Overview of the study protocol.

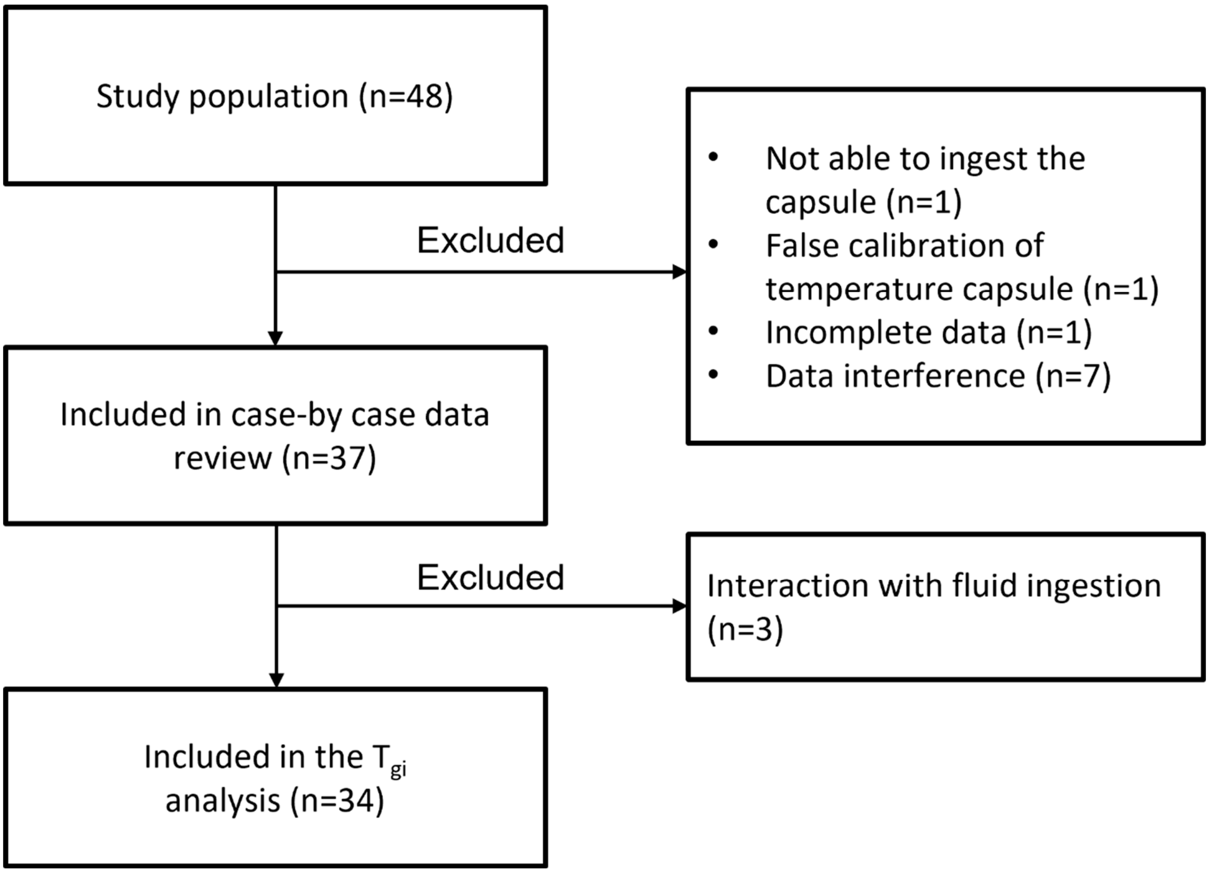

Supplementary Figure S2. Overview of the case-by-case review process of the T_{gi} data
